# The burnout phenomenon: Association and risk factors among dermatology residents in Gulf Cooperation Council countries

**DOI:** 10.1016/j.jdin.2024.07.021

**Published:** 2024-08-23

**Authors:** Dalal A. Aldosari, Asem M. Shadid, Mohammed N. Alabdullah, Sabika Alwazzan, Reem A. Alneyadi, Ahmed M. Alhinai, Mohammed Almashali, Ruaa Alharithy

**Affiliations:** a Department of Dermatology, King Saud Medical City, Riyadh, Saudi Arabia; b Department of Dermatology, King Fahad Medical City, Riyadh, Saudi Arabia; c Department of Dermatology, Hamad Medical Corporation, Doha, Qatar; d Department of Dermatology, Kuwait Institute for Medical Specialization, Andalous, Kuwait; e Department of Dermatology, Sheikh Khalifa Medical City, Abu Dhabi, UAE; f Department of Dermatology, Oman Medical Specialty Board, Muscat, Oman; g Assistant Professor and Dermatology Consultant, Dermatology Department, College of Medicine, Imam Mohammed ibn Saud Islamic University, Riyadh, Saudi Arabia; h Dermatology Consultant and Assistant Professor, Princess Nourah Bint Abdul Rahman University, Security Forces Hospital, Riyadh, Saudi Arabia

**Keywords:** Bahrain, burnout, depersonalization, dermatology resident, emotional exhaustion, GCC countries, Kuwait, Oman, personal accomplishment, Saudi Arabia, Qatar, United Arab Emirates

*To the Editor:* Burnout syndrome is characterized by emotional exhaustion (EE), depersonalization (DP), and reduced personal accomplishment (PA).^1^ It poses a public health concern, as it may progress to clinical depression, suicidal attempts, or ideation.^2^^,^^3^ More critical consequences include substance abuse, negligence, malpractice, and medical errors, all of which will lead to decreased patient safety and suboptimal patient care.^3^ Limited studies have been conducted nationally in the Middle East.^3^^,^^4^ A worldwide US study assessed the changes in burnout and other measures and found that burnout in dermatologists was higher in 2017 than in 2011, even though it was lower in 2014.^5^ Despite this interest, no further research has dug deeper into the issue. From our perspective, this gap in knowledge serves as sufficient justification to establish our research, which aims to estimate the prevalence of burnout and its associated risk factors in dermatology residents in Gulf Cooperation Council (GCC) countries.

A quantitative, observational, cross-sectional study was conducted among all dermatology residents on the boards of dermatology in Oman (*n* = 16), Kuwait (*n* = 36), Qatar (*n* = 13), and the UAE (*n* = 17) with a response rate of 90.2%. Maslach Burnout Inventory-Human Services Survey was distributed and a questionnaire assessing potential risk factors. Two-dimensional burnout was defined as the coexistence of high EE and high DP, whereas three-dimensional burnout was defined as low PA, high EE, and high DP.

Binary logistic regression was used to assess the factors associated with burnout dimensions and overall burnout. Odds ratios were calculated for each factor and tested for statistical significance using univariate binary logistic regression.

A high risk of EE and DP was predicted in 13.5% of residents (Table I). The risk of low PA was predicted in 27% of participants. The average EE score was higher among Qatari residents (33.3%) (*P* = .011). The average PA score was lower among Kuwait residents (39.4%) (*P* = .002), whereas the average DP score was not significantly different between the training programs. EE and feelings of low PA were observed at a high rate among the participants, whereas DP was the least prevalent across the three burnout dimensions. The risk of DP was significantly higher in married residents than in nonmarried residents (*P* = .056). Smoking was associated with a higher risk of EE (*P* = .029). A high risk of burnout was associated with lower satisfaction with salary (*P* = .039), career (*P* < .001), and work-life balance (*P* = .027). The prevalence of burnout did not differ significantly between countries (Fig 1); however, the overall risk of burnout in GCC countries (5.41%) was much lower than that in Saudi Arabia (21.4%).^4^ Three-dimensional burnout in dermatology residents in GCC countries is 1.4%.

**Table I:** Prevalence of burnout in the included residents

	[ALL]	Kuwait	Oman	Qatar	UAE	*P* overall
	*N = 74*	*N = 33*	*N = 16*	*N = 9*	*N = 16*	
Emotional exhaustion	16.7 (10.4)	18.3 (12.1)	9.50 (5.37)	21.8 (8.26)	17.9 (8.51)	**.011**
Depersonalization	3.78 (4.92)	5.21 (6.41)	1.94 (1.57)	3.89 (4.54)	2.62 (2.70)	.113
Personal accomplishment	36.4 (7.68)	33.9 (8.05)	42.3 (4.66)	34.1 (9.33)	36.7 (5.17)	**.002**
High emotional exhaustion:						.067
No	64 (86.5%)	27 (81.8%)	16 (100%)	6 (66.7%)	15 (93.8%)	
Yes	10 (13.5%)	6 (18.2%)	0 (0.00%)	3 (33.3%)	1 (6.25%)	
High depersonalization:						.281
No	69 (93.2%)	29 (87.9%)	16 (100%)	8 (88.9%)	16 (100%)	
Yes	5 (6.76%)	4 (12.1%)	0 (0.00%)	1 (11.1%)	0 (0.00%)	
Low personal accomplishment:						.067
No	54 (73.0%)	20 (60.6%)	15 (93.8%)	6 (66.7%)	13 (81.2%)	
Yes	20 (27.0%)	13 (39.4%)	1 (6.25%)	3 (33.3%)	3 (18.8%)	
Burnout (2-dimensional):						.312
No	70 (94.6%)	30 (90.9%)	16 (100%)	8 (88.9%)	16 (100%)	
Yes	4 (5.41%)	3 (9.09%)	0 (0.00%)	1 (11.1%)	0 (0.00%)	
Burnout (3-dimensional):						1.000
No	73 (98.6%)	32 (97.0%)	16 (100%)	9 (100%)	16 (100%)	
Yes	1 (1.35%)	1 (3.03%)	0 (0.00%)	0 (0.00%)	0 (0.00%)	

Data were summarized using counts and percentages.

Statistical analysis was performed using chi-square test of independence.

Bold values indicate *P* < .05.

**Fig 1:**
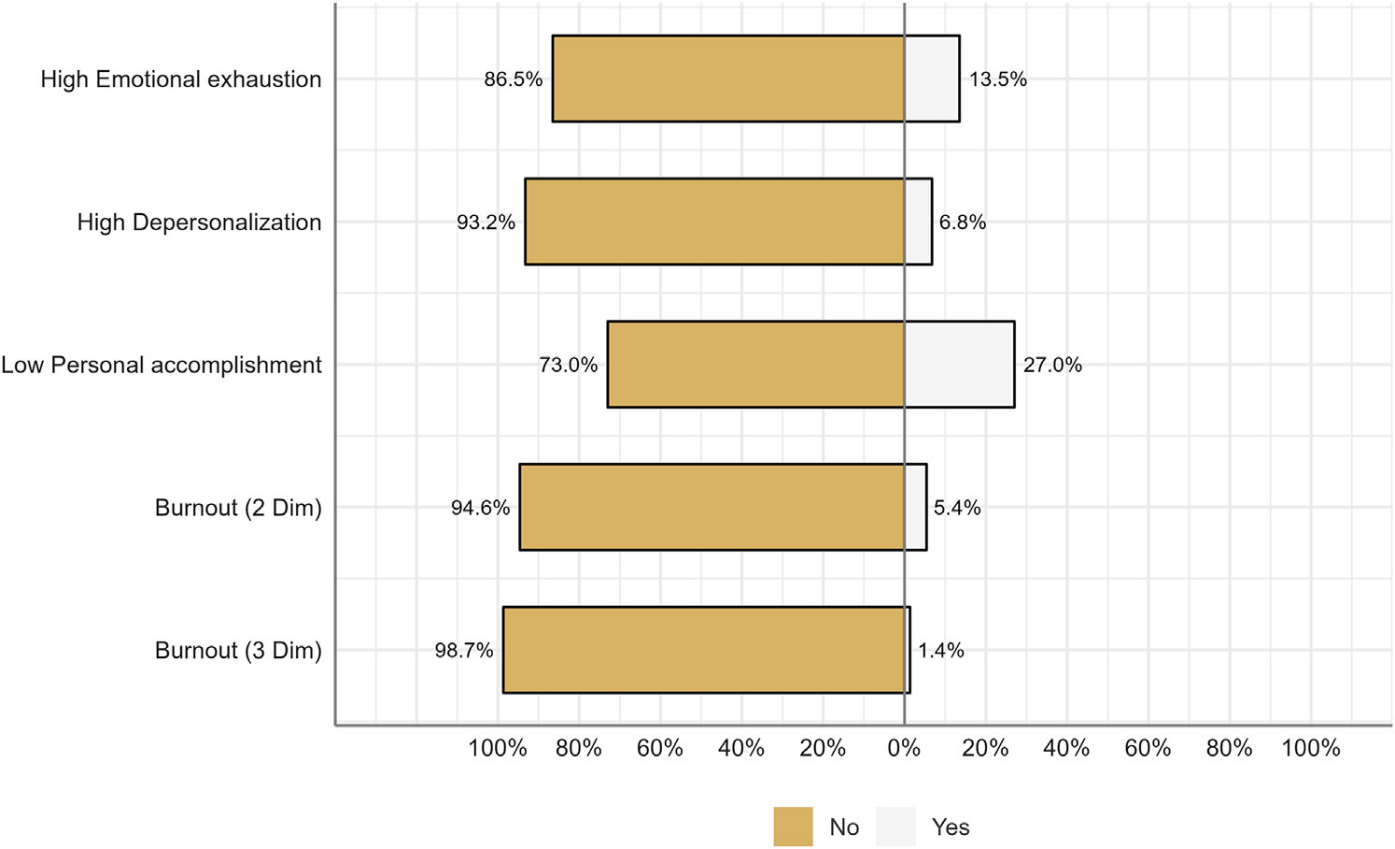
Prevalence of 2- and 3-dimensional burnout and high risk of EE, high risk of DP, and low risk of PA. *DP*, Depersonalization; *EE*, emotional exhaustion; *PA*, personal accomplishment.

Fair-on-call schedules, reasonable working hours, and a well-distributed number of clinics per week were considered contributing factors to the low level of burnout. Residents need better coping skills to enhance their well-being, increase their engagement at work, and minimize their burnout symptoms. We advocate raising burnout awareness in dermatology training programs and incorporating instruction and interventions into resident physician training.

## Conflicts of interest

None disclosed.
